# Field Tests of Three Alternative Insecticides with Protein Bait for the Development of an Insecticide Rotation Program to Control Melon Flies, *Zeugodacus cucurbitae* (Coquillett) (Diptera: Tephritidae)

**DOI:** 10.3390/insects13070629

**Published:** 2022-07-14

**Authors:** Ikkei Shikano, Rosemary Gutierrez-Coarite, Christian Streit, Edwin Perez, Earl Fujitani, Ronald F. L. Mau

**Affiliations:** 1 Department of Plant and Environmental Protection Sciences, University of Hawaii at Manoa, Honolulu, HI 96822, USA; cstreit@hawaii.edu (C.S.); maur@ctahr.hawaii.edu (R.F.L.M.); 2 Kahului Extension Office, Department of Tropical Plant and Soil Sciences, University of Hawaii at Manoa, Kahului, HI 96732, USA; gr6@hawaii.edu; 3 Maui Agricultural Research Center, University of Hawaii at Manoa, Kula, HI 96790, USA; eperez@hawaii.edu (E.P.); efujitan@hawaii.edu (E.F.)

**Keywords:** Cucurbitaceae, GF-120, insecticide resistance, integrated pest management, protein bait, Tephritidae fruit fly

## Abstract

**Simple Summary:**

Melon flies in Hawaii have developed high levels of resistance to a widely used insecticidal bait containing the active ingredient spinosad. Since farmers relied heavily on this product and have few alternatives, we tested three other insectides mixed with a bait spray called Nu-Lure. The three products (Agri-Mek SC, Mustang Maxx, and Malathion 5EC) were all found to be effective in either decreasing melon fly numbers or suppressing the growth of the population in the field. When used one-by-one in rotation for two weeks per product at one-week intervals on a commercial zucchini farm, melon fly populations were dramatically reduced and the yield of zucchinis harvested was substantially increased.

**Abstract:**

High levels of resistance to the spinosad-based insecticidal protein bait GF-120 have been detected in some populations of melon fly, *Zeugodacus cucurbitae* (Coquillett) (Diptera: Tephritidae), in Hawaii in 2017. To provide cucurbit farmers in Hawaii with alternative insecticides, we field-tested the effectiveness of Agri-Mek SC (a.i., abamectin), Mustang Maxx (a.i., zeta-cypermethrin), and Malathion 5EC (a.i., malathion), added to a protein bait spray (Nu-Lure Insect Bait). The insecticide and protein bait combinations were applied to the roosting plants of *Z. cucurbitae* around the perimeter of the cucurbit fields at one-week intervals. When individually tested, all three insecticides in combination with protein bait significantly reduced or suppressed the numbers of female flies caught in torula yeast traps. A two-week rotation of weekly applications of the three insecticides and GF-120 significantly reduced *Z. cucurbitae* numbers on a commercial zucchini farm on Maui. The percentage of marketable fruits harvested increased from 51% to 98% after implementing the insecticide rotation. Our findings will be used to provide cucurbit farmers with additional products to control *Z. cucurbitae*. The future focus will be on educating cucurbit farmers to use the insecticide rotation strategy to prevent or delay resistance development.

## 1. Introduction

The melon fly, *Zeugodacus cucurbitae* (Coquillett) (Diptera: Tephritidae), is a devastating agricultural pest that has invaded many temperate, tropical, and sub-tropical regions of the world [1]. Its preferred host are young, developing fruits of plants in the family Cucurbitaceae, but it is believed to be capable of infesting fruits in at least 136 plant taxa across 62 genera [2]. *Zeugodacus cucurbitae* is usually only seen on the host crops when the females are ovipositing in the fruits. Males, reproductively immature females, and mature females that are not actively ovipositing spend most of their time aggregating on non-host plants, termed roosting plants, which are usually present in the perimeter of the host crop fields [3,4]. The roosting plants serve as lekking sites [5,6], provide shade, sugar sources from flowers and extrafloral nectaries [4,7], and protein sources from pollen, bird droppings, and others. [8,9]. Because of a greater comparative presence of *Z. cucurbitae* on the roosting plants than crop plants at most times of the day, management practices in Hawaii and some other regions focus on targeted insecticide and protein bait treatments on the roosting plants rather than full coverage sprays on crops [10,11,12].

The mild climate in Hawaii and high local market demand for cucurbit fruits allow for year-round production of cucurbit crops, which creates optimal conditions for *Z. cucurbitae* reproduction. It is estimated that there are more than 10 generations of *Z. cucurbitae* per year [13,14]. In 2001, GF-120^®^ NF Naturalyte^®^ Fruit Fly Bait (Dow AgroSciences, Indianapolis, IN, USA), which is a sprayable protein bait containing spinosad as the active ingredient, was introduced to Hawaii. GF-120 quickly became the most widely used and often only control method used to control *Z. cucurbitae* in Hawaii. It is applied as targeted applications to the roosting plants [11,15,16]. Continuous year-round use of GF-120 has placed high selection pressure on *Z. cucurbitae* populations. A survey of spinosad resistance conducted in Hawaii in 2017 revealed up to 300-fold higher resistance to spinosad in melon fly populations collected from commercial farms on Oahu compared to a susceptible lab population [17]. The resampling of one of the Oahu populations one year after cessation of GF-120 use revealed that resistance persisted, albeit at a lower level (25-fold) [17]. A lower level of spinosad resistance was also found in a Maui population (8.5-fold) [17]. The evolution and persistence of high levels of resistance to spinosad in melon flies highlights the need for alternative control tactics.

Insecticide resistance management aims to slow or prevent resistance evolution and also to revert a resistant pest population to susceptibility by reducing the selection pressure from a particular insecticide on the target pest population [18]. One widely used approach is to rotate the use of different insecticides while simultaneously restricting the duration that each insecticide is used. The rotation is typically conducted using compounds in different mode-of-action groups. Compounds in the same group, including those in different subgroups, are avoided because of the higher probability of selecting for a common target site-based resistance mechanism [18]. The length of time an insecticide is used is usually set to the length of the pest’s life cycle or a particular growth stage of the crop [18]. The insecticide rotation approach was implemented in Hawaii in 2001 to manage insecticide resistance in the diamondback moth, *Plutella xylostella* [19], and remains in effect today.

Here, we describe field experiments that were conducted to test the efficacy of three alternative insecticides to spinosad in different IRAC mode-of-action groups (Table 1): Agri-Mek SC (a.i., abamectin; Syngenta Crop Protection LLC, Greensboro, NC, USA), Mustang Maxx (a.i., zeta-cypermethrin; FMC Corporation, Philadelphia, PA, USA), and Malathion 5EC (a.i., malathion; Drexel Chemical Company, Memphis, TN, USA), added to a protein bait spray (Nu-Lure Insect Bait; Miller Chemicals & Fertilizer LLC., Hanover, PA, USA). Agri-Mek SC, Mustang Maxx, and Malathion 5EC are registered for use on cucurbit field crops (EPA crop groups 9a and 9b). Before the introduction of spinosad, malathion was the most common insecticide additive to protein baits due to its low mammalian toxicity, affordable price, and low levels of fruit fly resistance [20]. Abamectin has fewer negative effects on nontarget insects than organophosphate insecticides [21] and is effective against *Z. cucumis*, several *Bactrocera* and *Anastrepha* species [22,23,24,25,26,27,28,29]. Zeta-cypermethrin has been shown to reduce oviposition by *Rhagoletis indifferens* on cherries [30]. All three insecticide products were confirmed to be effective as oral and contact toxins to *Z. cucurbitae* in laboratory assays prior to use in the present study (Hsu et al., unpublished). The goal of the study was to develop a rotation of insecticides, consisting of GF-120 and the three alternative insecticides, to preserve their long-term efficacy. To determine the efficacy of individual insecticides, we conducted spot spray applications of Nu-Lure with each insecticide to treat border plants on the perimeter of the cucurbit crops. These treated plants included naturally occurring known roosting plants (e.g., castor bean, jackfruit, haole koa) and intentionally planted roosting plants, including Sudex (i.e., sorghum sudangrass hybrid), eggplant, and long beans. The insecticides were not applied to the cucurbit crop. We then tested the effectiveness of a rotation of insecticides, consisting of GF-120 and the three alternative insecticides, on suppressing the melon fly population and marketable fruit yield.

## 2. Materials and Methods

### 2.1. Nu-Lure and Insecticide Application

Agri-Mek SC, Mustang Maxx, and Malathion 5EC were each tested in combination with Nu-Lure protein bait within maximum label rates (Table 2). The concentrations of products used in each experiment are listed in Table 3 and Table 4. Ammonium acetate was added to the bait-insecticide mixture as it was previously shown to improve attractiveness of the spray to melon flies and other tephitid fruit flies [31,32,33,34]. The percentage of ammonium acetate in the final mixed solution is listed in Table 3 and Table 4. We followed application procedures in the University of Florida IFAS Extension’s Fruit Fly Bait Spray Guide. Inverted bottle traps containing Cue-lure plugs (Scentry Biologicals Inc., Billings, MT, USA) and multi lure McPhail-type traps baited with 3 torula yeast tablets (Scentry Biologicals Inc., Billings, MT, USA) diluted in 300 mL of water per trap were used to attract melon flies and to identify roosting plants prior to spot spray treatments. Cue-lure is a male attractant [35] and torula yeast attracts both males and females [36]. The Nu-Lure and insecticide combination was sprayed at 7-day intervals as a low volume application using wand-type spot-spray equipment. Spot sprays were directed to the underside of the leaves on the inside of the canopy to reduce exposure of the treatments to sunlight and rainfall. We did not record temperature, weather, and rainfall during the field trials. However, there were no rain events on the day of insecticide applications and temperature remains mostly constant throughout the year in Hawaii, such that cucurbits are grown year-round.

### 2.2. Experimental Design

Melon flies are strong flyers, capable of single continuous flight bouts lasting more than 100 min [37,38]. Therefore, they can regularly move hundreds of meters between cropped areas and attractive roosting plants, as well as between fields/farms to seek oviposition sites [39]. This made the selection of field sites difficult as immigration of melon flies from surrounding farms could conceal the effects of our insecticide treatments. We conducted one trial of each insecticide in 2020 at a small commercial zucchini farm (<12 hectares) that is located at a high elevation of 460 m in Kula, Maui. The farm sits above several other cucurbit farms, which we believed would minimize immigration of melon flies to the farm. Additionally, we conducted a second Agri-Mek trial at a large commercial farm in Wahiawa, Oahu (>120 hectares). This farm grew mostly cucurbits (zucchini, melons, winter squash) and was surrounded by pineapple fields and non-agricultural land. Lastly, we conducted trials of the three insecticides at a UH CTAHR Research Station in Kula, Maui. The research station is surrounded by commercial cucurbit farms. Since our experimental zucchini plot was small and was the only cucurbits being grown at the research station, we relied on the neighboring farms for immigration of flies into our plot.

*2020 Maui Commercial Farm Field Trial:* The first field trial was conducted at a small commercial farm on Maui. The farm had a windbreak of trees down the middle, such that we were able to split the farm into treated and control sides on opposite sides of the windbreak. To monitor the abundance of melon flies, torula yeast traps were set up on four locations in the treated area and three locations in the control area (about 100 m distance between traps). The numbers of traps between treatment and control areas differed because we only used trap locations (i.e., plants) that were previously found to be consistently popular roosting sites for melon flies on this farm. Sprays of insecticide in Nu-Lure Insect Bait were conducted once per week for two consecutive weeks between 8 and 9 a.m. Known roosting plants and other potential roosting sites were spot-sprayed with 90 mL of the mixture (Table 3) using a 4-gallon piston backpack sprayer. The area within the treated roosting plants encompassed approximately 2 hectares (5 acres). Melon flies were collected weekly from the torula yeast traps immediately before insecticide applications, and the numbers of male and female melon flies were counted. To determine the effect of the insecticide applications, we obtained the baseline population numbers by collecting two weekly trap counts before Agri-Mek and Malathion applications and one trap count before Mustang Maxx applications. The effect of the applications was evaluated by dividing the number of female flies caught in each trap post-treatment by the pre-treatment trap count in the week prior to the first post-treatment trap count (i.e., the relative change in trap counts). 

For Agri-Mek, pre-treatment trap captures were collected between 7 and 8 a.m. on 21 July and 28 July. Then, the first Agri-Mek application was performed between 8 and 9 am on 28 July, followed by collection of the first post-treatment trap captures on 4 August, which was immediately followed by the second Agri-Mek application, and then finally, the second post-treatment trap captures were collected on 11 August. The malathion trial was initiated the week following the Agri-Mek trial. To ensure that any residual effect of Agri-Mek had dissipated, we conducted two pre-treatment trap counts (18 August and 25 August), which showed that fly populations were increasing on both the control and treated areas of the farm. Post-treatment trap captures were collected on 1 September, 8 September and 15 September. The malathion applications were performed after trap capture collections on 25 August, 1 September and 8 September. For Mustang Maxx, only one pre-treatment trap count was conducted (30 November), which was immediately followed by the first treatment application and a second application on 7 December. Post-treatment trap captures were collected on 7 December and 14 December.

*2020 Oahu Commercial Farm Field Trial*: The second field trial was conducted at a large commercial farm in central Oahu. Due to the size of the farm and labor required, only Agri-Mek SC was tested at this commercial farm. The farm had rows of jackfruit trees serving as windbreaks and roosting sites for melon flies. The treatment was applied to three windbreaks (580, 650, 750 m in length). Seven torula yeast traps were set up along the jackfruit windbreaks in the treated area; one trap in the shortest windbreak and three each in the longer two windbreaks. The area within the treated windbreaks encompassed approximately 39 hectares (96 acres). Three traps were set up in control areas at least 500 m away from the nearest treated windbreak. All the traps in the treated and control areas were placed adjacent to plots of cucurbit crops. 

A single spot spray of 90 mL (Table 2) was applied at 6.1 m (20 ft) intervals along the jackfruit windbreaks. The spot-spray equipment consisted of a battery-operated backpack sprayer with an adjustable nozzle (RYOBI 18-Volt ONE + Backpack Chemical Sprayer, Ryobi Limited, Fuchu, Japan). The sprayer had a 4-gallon tank and an 18 V lithium-ion battery that sprayed at a pressure of 60 PSI. Pre-treatment trap captures were collected between 7 and 8 a.m. on 28 July and 3 August. Then, the first Agri-Mek application was performed between 8 and 9 a.m. on 3 August, followed by collection of the first post-treatment trap captures on 10 August, which was immediately followed by the second Agri-Mek application, and then finally, the second post-treatment trap captures were collected on 17 August.

*2021 Maui Research Station Field Trial:* The third field trial was conducted on a plot at UH CTAHR Research Station in Kula, Maui. We tested GF-120 in addition to Agri-Mek, Mustang Maxx and Malathion. The plot consisted of three 12.2 m rows of zucchini surrounded on three sides by three 6.1 m rows of long beans, three 6.1 m rows of eggplant, and two 6.1 m rows of Sudex. Long beans were grown on a trellis. All three of these plants are known roosting plants for melon flies. Five-week-old zucchini, long bean, and eggplant were transplanted 4 weeks before each trial. Sudex was transplanted 6 weeks before each trial. New zucchini, long bean, eggplant, and Sudex were transplanted every three weeks in adjacent plots for the following trials. Plots were immediately destroyed after the second week of trap collections. For each trial, one torula yeast trap was set up in each of these roosting plant types, as well as one trap on a mango tree and one trap on a Haole Koa tree within the research station field. The intentionally planted roosting plants were sprayed in a swath along each row (Table 2). The mango and Haole Koa were large trees and, hence, were spot sprayed in various spots. Other potential roosting plants within the research station were also spot sprayed. There were no control areas in this trial. Instead, trap counts were compared before and after insecticide application. The torula yeast traps were set up 2 days prior to insecticide application to obtain a pre-treatment count, sprayed, and then traps were collected 2 days later to obtain a post-treatment count. Traps were then removed, and the process was repeated the following week. 

*2021 Insecticide Rotation Trial on Maui Commercial Farm:* An insecticide rotation trial was conducted on the commercial farm where the 2020 Maui Commercial Farm Field Trial was conducted. The experiment was initiated earlier than planned as requested by the farmer due to high infestation rates in his zucchini crop. At the start of the trial, only one trap was set up in the control area, and two additional traps were set up in the control area immediately after the first GF-120 application. Therefore, the “pre-treatment” count for two out of three traps in the control area were after the first insecticide treatment. Otherwise, the experimental design and trap placements were the same as the previous field trials at that farm with 4 insecticides used in rotation. Each insecticide (Table 3) was applied in one-week intervals for two weeks. On 29 April, the first GF-120 Naturalyte Fruit Fly Bait (1:1.5 in water) application was made immediately following the pre-treatment count from the torula yeast traps. The effectiveness of the treatment was assessed by collecting and counting the numbers of flies in the traps the following week (6 May). This trap collection was immediately followed by another GF-120 application, and followed by applications of Agri-Mek + Nu-Lure on 13 May and 20 May, Mustang Maxx + Nu-Lure on 27 May and 3 June, Malathion + Nu-Lure on 10 June and 17 June, GF-120 again on 24 June and 1 July, and Agri-Mek + Nu-Lure on 8 July and 15 July. 

To estimate the effectiveness of the insecticide rotation on fruit yield, the farmer recorded the numbers of buckets of marketable and infested fruits harvested each week for nine consecutive weeks. Although we were unable to obtain data on numbers and weight of harvested fruits, the buckets used by the farmer were all uniform in size (4-gallon buckets). This was a compromise we made to collect harvest data without impeding daily farm procedures. 

### 2.3. Statistical Analyses

Only traps that caught at least 10 female flies during the pre-treatment count were included in statistical analyses because the effects of the insecticide treatment could not be reliably measured with lower pre-treatment counts. For the Maui and Oahu commercial farm field trials and the insecticide rotation, the data were analyzed as the relative change in the numbers of female flies caught in the torula yeast traps post treatment. The data in all field trials were analyzed by repeated measures (mixed model) analysis of variance (ANOVA) with treatment, date and their interaction included as fixed factors and the trap as a random factor. Non-significant interactions were removed to produce the final minimal model. Means contrasts were performed to compare the numbers of flies caught in control and treated areas even when the interaction term (treatment × date) was not significant because the effects of insecticides on the trap catches changes with the number of sequential applications. The degrees of freedom values from the mixed model that were in decimals are presented in the nearest whole number. The relative number of flies in the traps were log transformed. The number of female flies in the research station trial was square root transformed to meet the assumptions of normality. The mean percent of marketable fruits per week from the insecticide rotation experiment was analyzed by repeated measures (mixed model) ANOVA with treatment (treated area vs. control area) as the fixed factor and date as a random factor. The dates of 29 April and 10 June were excluded from analyses due to lack of data. All statistical analyses were performed on JMP Pro 16 (JMP Statistical Discovery LLC, Cary, NC, USA).

## 3. Results

### 3.1. Agri-Mek SC with Nu-Lure

**Figure 1:**
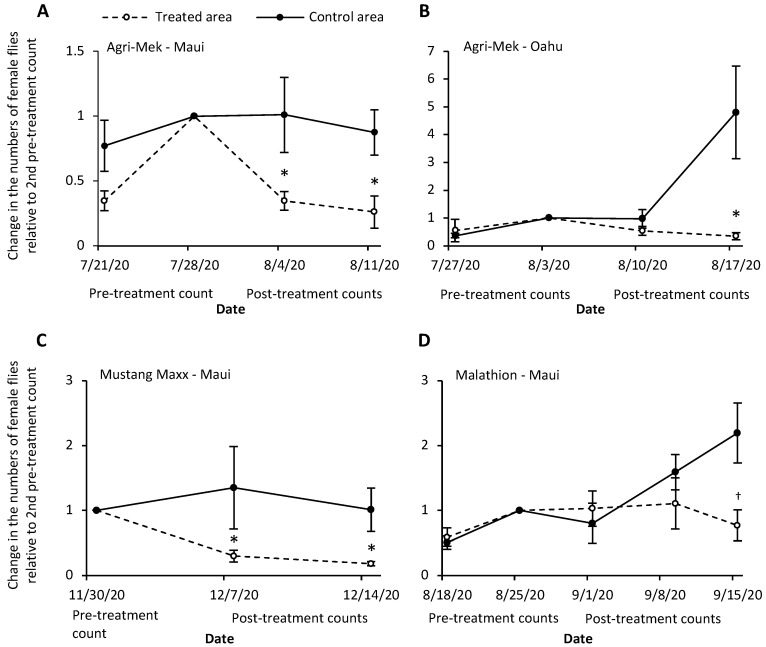
The change in the numbers of female *Zeugodacus cucurbitae* caught in torula yeast traps relative to the pre-treatment count in the week prior to the first post insecticide-treatment count. (**A**) Agri-Mek SC Maui commercial farm trial; (**B**) Agri-Mek SC Oahu commercial farm trial; (**C**) Mustang Maxx Maui commercial farm trial; (**D**) Malathion 5EC Maui commercial farm trial). The dates (m/d/y) refer to the date that traps were collected and counted; the insecticide was applied one week prior to the post-treatment count date. Grey line represents no change from pre-treatment count. Asterisk above treated area symbols indicates significant difference between control and treated areas based on means contrasts for each date (* *p* < 0.05, ^†^ *p* < 0.10).

**Figure 2:**
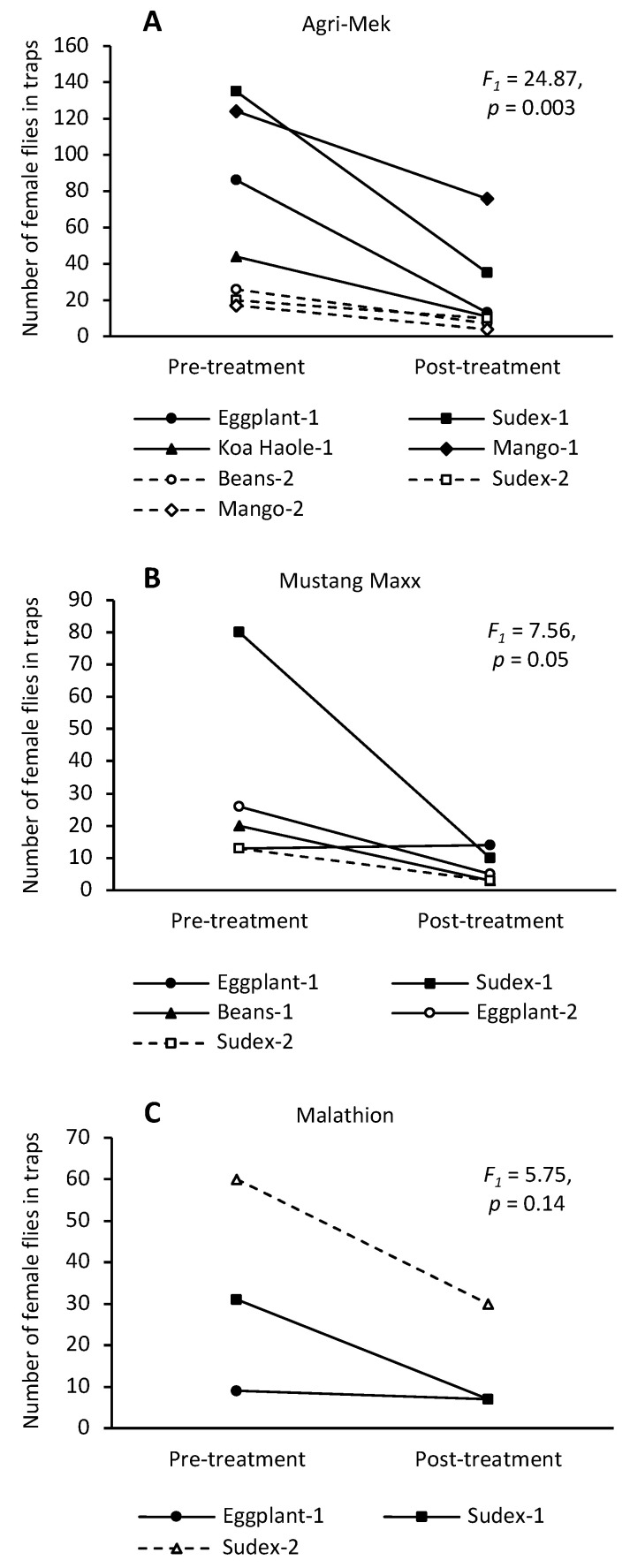
The numbers of female *Zeugodacus cucurbitae* caught in torula yeast traps on roosting plants in the two days prior to insecticide application and two days after application at the Maui research station ((**A**) Agri-Mek SC; (**B**) Mustang Maxx; (**C**) Malathion 5EC).

In the 2020 Maui commercial farm field trial, three traps in the treated area and two traps in the control area captured at least 10 female flies during the second pre-treatment count. Agri-Mek treatment decreased the number of female flies in the traps in the two weeks following applications, while the number of flies caught did not change significantly in the control area (Figure 1A). This resulted in significant differences in relative female trap catches between control and treated areas in the two weeks following Agri-Mek sprays (means contrast: 4 August 2020, *p* = 0.04; 11 August 2020, *p* = 0.05) (treatment: *F_1,3_* = 10.36, *p* < 0.05; date: *F_1,4_* = 1.67, *p* = 0.27; treatment × date: *F_1,3_* = 0.04, *p* = 0.81). In the 2020 Oahu field trial, all traps in the treated areas (seven traps) and control areas (three traps) contained at least 10 female flies on the second pre-treatment count. Agri-Mek treatment suppressed the number of female flies caught in the traps relative to the pre-treatment catches (Figure 1B). However, the effect of the treatment was only evident after the second application (means contrast: 10 August 2020, *p* = 0.94; 17 August 2020, *p* < 0.001) (treatment: *F_1,8_* = 5.90, *p* = 0.04; date: *F_1,8_* = 17.72, *p* = 0.003; treatment × date: *F_1,8_* = 59.24, *p* < 0.0001). At the UH CTAHR research station on Maui in 2021, seven traps caught at least 10 female flies during the pre-treatment period. Two days after spraying, significantly fewer flies were caught in the traps (treatment: *F_1,6_* = 24.87, *p* = 0.003; Figure 2A).

### 3.2. Mustang Maxx with Nu-Lure

In the 2020 Maui commercial farm field trial, three traps in the treated area and three traps in the control area captured at least 10 female flies during the lone pre-treatment count. The number of female flies in the traps decreased in the area treated with Mustang Maxx but not in the control area (Figure 1C). This resulted in significant differences in relative female trap catches between control and treated areas in the two weeks following Mustang Maxx sprays (means contrast: 07 December 2020, *p* = 0.02; 14 December 2020, *p* = 0.008) (treatment: *F_1,4_* = 8.80, *p* = 0.02; date: *F_1,5_* = 0.94, *p* = 0.39; treatment × date: *F_1,4_* = 0.14, *p* = 0.73). At the UH CTAHR research station, five traps caught at least 10 female flies during the pre-treatment period. Two days after spraying, fewer flies were caught in four of the traps, although the effect was only marginally significant (treatment: *F_1,4_* = 7.56, *p* = 0.05; Figure 2B).

### 3.3. Malathion 5EC with Nu-Lure

In the 2020 Maui commercial farm field trial, all traps in the treated area (four traps) and control area (three traps) captured at least 10 female flies during the second pre-treatment count. Application of Malathion did not decrease the number of female flies in the traps relative to the pre-treatment catches. However, three weekly applications prevented an increase in the fly population compared to the control area (Figure 1D). This resulted in a marginally significant difference in relative females catches between the treated and control areas after the third application, but not after the first two applications (means contrast: 1 September 2020, *p* = 0.61; 8 September 2020, *p* = 0.27; 15 September 2020, *p* = 0.06) (treatment: *F_1,7_* = 0.28, *p* = 0.61; date: *F_2,10_* = 2.54, *p* = 0.13; treatment × date: *F_2,10_* = 7.12; *p* = 0.01). At the UH CTAHR research station, only three traps caught at least 10 female flies during the pre-treatment period. While counts in all three traps were lower after application, this was not statistically different from the pre-treatment counts (treatment: *F_1,2_* = 5.75, *p* = 0.14; Figure 2C).

### 3.4. Insecticide Rotation Field Trial

**Figure 3:**
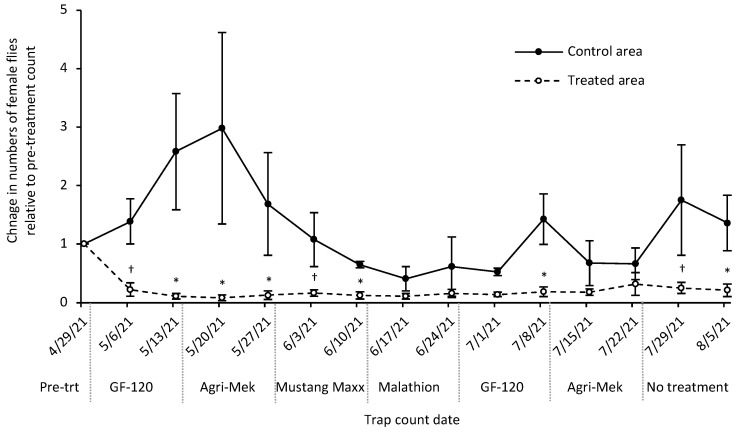
The change in the numbers of female *Zeugodacus cucurbitae* caught in torula yeast traps relative to the pre-treatment count during the insecticide rotation experiment on the Maui commercial farm. The dates (m/d/y) refer to the date that traps were collected and counted; the insecticide was applied one week prior to the trap count date. Grey solid line represents no change from pre-treatment count. Asterisk above treated area symbols indicate a significant difference between control and treated areas based on means contrasts for each date (* *p* < 0.05, ^†^ *p* < 0.10).

In the 2021 insecticide rotation trial on a Maui commercial zucchini farm, three traps in the treated area and two traps in the control area captured at least 10 female flies during the lone pre-treatment count. After the first application of GF-120, the numbers of female flies caught in the traps decreased in the treated area and increased in the control area (Figure 3; Supplementary Table S1). Overall, the numbers of female flies in the treated area remained suppressed until two weeks after the termination of the insecticide rotation treatments. In the control area, the numbers of flies increased for about 4 weeks and then decreased, likely because the treatments in the treated area reduced the overall populations of flies on the farm (treatment: *F_1,3_* = 11.56, *p* = 0.04; date: *F_13,52_* = 0.95, *p* = 0.51; treatment × date: *F_13,39_* = 1.23, *p* = 0.30). The percentage of female flies in the traps were similar between control (55% female) and treated areas (52% female) during the first trap count, but this diverged to become more male-biased in the treated areas over the course of the experiment with an average of 63% females in the control area and 43% in the treated area from 6 May 2021 to 5 August 2021 (treatment: *F_1,3_* = 6.01, *p* = 0.09; date: *F_13,52_* = 2.37, *p* = 0.01; treatment × date: *F_13,39_* = 1.19, *p* = 0.33), which suggests that the insecticide-bait combination was having a marginally greater effect on the female melon flies. The lowest percentage of females in the treated area was 28% (10 June 2021) while the lowest in the control area was 46% (1 July 2021).

The percentage of marketable fruits harvested by the farmer was approximately 51–53% at the start of the insecticide rotation. This increased to 85% after four insecticide applications in the treated area and almost all fruits were harvestable after three more applications (Table 5). The percentage of harvestable yield in the control area increased gradually over time, likely as the overall numbers of flies on the farm decreased from insecticide applications in the treatment area. Overall, the mean percent of marketable fruit yield per week was significantly higher in the treated area (81%) than in the control area (62%) (treatment: *F_1,7_* = 13.33, *p* = 0.008).

## 4. Discussion

Insecticide applications to the roosting plants of *Z. cucurbitae* in the perimeter of cucurbit crops resulted in dramatically lower volumes of each insecticide being used than the maximum label rates for direct application to the crop, with the added benefit of no insecticide residues on the crop. Individual field tests of Agri-Mek SC, Mustang Maxx, and Malathion 5EC mixed in Nu-Lure Insect Bait at high and low concentrations suggest that they were effective in reducing or suppressing *Z. cucurbitae* populations. Malathion was the only active ingredient that did not significantly reduce the numbers of *Z. cucurbitae* captured in traps relative to the pre-treatment counts, though it appears to have prevented an increase in the numbers of *Z. cucurbitae* in the 2020 trial. Aside from spinosad, the only other insecticide that is registered in Hawaii for use in mixes with protein bait is naled (Diabrom), which is in the same IRAC mode-of-action subgroup as malathion. Since farmers were recommended to cease the use of spinosad-based products to control *Z. cucurbitae* in 2017, those with licenses to use restricted-use pesticides have been dependent on naled. Thus, an overreliance on naled by some farmers could lead to the development of naled-resistance in *Z. cucurbitae*, and possibly cross-resistance to malathion, in some *Z. cucurbitae* populations in Hawaii. The mechanism of malathion resistance in Oriental fruit flies (*Bactrocera dorsalis*) and *Ceratitis capitata* (Mediterranean fruit flies) is associated with esterase detoxification [40,41,42]. Cross-resistance between malathion and naled has been detected in lab selection experiments on *B. dorsalis* [43] and malathion-resistant *C. capitata* have been shown to exhibit high levels of cross-resistance to other organophosphates [41]. To the best of our knowledge, the commercial farm on which we conducted our malathion trial had never used naled to control *Z. cucurbitae*. Another possibility is the exposure of *Z. cucurbitae* to malathion on roosting plants that are treated to control other pests. Malathion is a common insecticide used to control aphids, whiteflies and spider mites in vegetable and fruit farms in Hawaii. Several known roosting plants of *Z. cucurbitae*, such as beans and eggplants, are constantly under attack by whiteflies, thrips, and spider mites, and hence, regularly treated with malathion. Lastly, the addition of malathion to protein baits, including Nu-Lure, has been shown to repel and deter consumption by *Z. cucurbitae* [44] and other Tephritidae species [45,46]. Thus, *Z. cucurbitae* may have consumed less of the malathion bait compared to the other insecticide-bait mixtures in our trials. Further research is needed to determine the appropriate concentration of Malathion 5EC in protein bait spray to facilitate ingestion by *Z. cucurbitae*.

As strong levels of spinosad resistance were detected in some *Z. cucurbitae* populations in Hawaii, the potential for cross-resistance to spinosad and the alternative insecticides could be a concern. In *Spodoptera exigua* with high levels of spinosad resistance (RR = 345), no cross-resistance between spinosad and abamectin, fenvalerate, cyfuthrin, phoxim, and methomyl was detected [47]. However, lab-selected spinosad-resistant *Z. cucurbitae* (RR = 35.79) exhibited resistance to beta-cypermethrin (RR = 11) and low level resistance to avermectin (RR = 5.45) and no cross-resistance to trichlorfon and fipronil [48]. Our field sites on Maui was in very close proximity to where spinosad resistance (RR = 8.5) was detected in 2017 [17]. Resistance in that Maui population reverted to susceptibility within 6 generations in the absence of spinosad exposure in the lab. Therefore, while we did not check the spinosad resistance of the field populations prior to our field treatments, the *Z. cucurbitae* at the Maui field sites were likely susceptible to spinosad at the time of our field trials. The spinosad-resistance of the *Z. cucurbitae* at our Oahu field site was not tested in the previous study.

The timing of our insecticide rotation was selected to minimize possibilities of resistance evolution. Based on our rotation schedule, the duration of one insecticide is 14 days and the full rotation of four insecticides is 56 days. In the typical temperature range of cucurbit farms in Hawaii, *Z. cucurbitae* develop from egg to eclosion of the adult fly in about 18.1–12.9 days (24–32 °C, respectively). Therefore, the insecticide encountered by a newly eclosed melon fly is unlikely to be the same as that encountered by its parent. Moreover, the longevity of adult melon fly females under laboratory conditions ranges widely from 15 to 79 days depending on their access to food and host fruits [49,50]. In the field, adult longevity is likely reduced due to numerous mortality factors, such as predation, disease, and desiccation. In our 2-week rotation, any flies that survive one insecticide will encounter another class of insecticide during its adult life, which should limit the oviposition period of individuals resistant to one of the insecticide classes. Our complete insecticide rotation was clearly successful in decreasing and suppressing *Z. cucurbitae* numbers on the treated side of the farm. This corresponded to a much higher percentage of marketable fruits harvested until the cessation of the insecticide applications. The insecticide rotation approach has been successfully used in Hawaii since 2001 to control *Plutella xylostella* (diamondback moth) on cruciferous vegetable farms [19]. It was readily adopted and remains effective to this day. The approach has also been successful in controlling *Helicoverpa armigera* in Australia [51] and Asian citrus psyllid, *Diaphorina citri* in Florida [52]. 

Our study demonstrated that applications of Agri-Mek SC, Mustang Maxx, and Malathion 5EC with Nu-Lure protein bait in two-week rotations to roosting hosts of *Z. cucurbitae* can effectively decrease and keep populations suppressed. The application method required no treatments on the cash crop and the amount of active ingredients used was far below the label limits. Importantly, to be able to measure the effect of our treatments, we intentionally conducted our field trials on farms that would have lower levels of immigration of melon flies from surrounding areas. Since melon flies are strong flyers [37,38], the use of these insecticides in a rotation on most farms would be most effective if used in an area-wide approach. Another important consideration, especially with widespread or areawide use, is the susceptibility of nontarget species to the insecticides, including bees and natural enemies of pests. All of the insecticides we tested can be harmful to nontarget species and beneficial insects (abamectin [53,54], malathion [55,56], and zeta-cypermethrin [57,58]), including GF-120 at high density application rates [56]. The protein in Nu-Lure Insect Bait might also attract nontarget species. Green lacewings have been shown to feed on Nu-Lure in the laboratory [59], but not the parasitoid *Fopius arisanus*, which is a major natural enemy of *C. capitata* in Hawaii [60]. While applications of the bait-insecticide sprays on roosting plants of *Z. cucurbitae* will limit the exposure of nontarget species to the insecticides compared to full coverage crop sprays, applications to roosting plants in bloom, areas near aquatic habitats, and areas where threatened and endangered species are known to occur should be avoided. Our next steps are to obtain local registrations for the off-label use of these insecticidal products, to educate farmers to use the products in rotation to prevent or slow-down resistance development, and develop an insecticide rotation schedule to be used area-wide in major cucurbit growing regions in Hawaii. 

## Figures and Tables

**Table 1:** IRAC Mode of Action classification for the insecticide active ingredients (abamectin, zeta-cypermethrin, and malathion) that were field-tested against *Zeugodacus cucurbitae* mixed in Nu-Lure Insect Bait.

Brand Name (Common Name)	Active Ingredient	IRAC Group/Subgroup	Primary Site of Action/Mode of Action (Based on IRAC MoA Classification)
Nu-Lure	-	-	-
Agri-Mek SC	8% abamectin	6 (Avermectins and milbemycins)	Chloride channel activator
Mustang Maxx	9.15% zeta-cypermethrin	3B (DDT and analogs)	Voltage gated sodium channel modulator
Malathion 5EC	57% malathion	1B (organophosphates)	Acetylcholinesterase inhibitor

**Table 2:** The maximum label use rates for Agri-Mek SC, Mustang Maxx, and Malathion 5EC when applied directly to cucurbit crops to control various insect pests in Hawaii. The present study applied the insecticides (mixed with Nu-Lure Insect Bait) to roosting plants in the perimeter of cucurbit crop fields to control *Zeugodacus cucurbitae*.

	Ground Sprayer				
Brand Name (Common Name)	Amount (fl oz/acre)	Amount of Water (gal Water/acre)	mL of Product/L Water	g/L of Active Ingredient	g of Active Ingredient/acre
Nu-Lure	48	10	37.5	-	-
Agri-Mek SC	3.5	20	1.37	0.115	8.68
Mustang Maxx	4	10	3.125	0.30	11.34
Malathion 5EC	44.8	10	35.0	20.97	793.79
	**Aerial sprayer**				
Nu-Lure	48	1	375	-	-
Agri-Mek SC	3.5	5	5.48	0.46	8.68
Mustang Maxx	4	2	15.63	1.50	11.34
Malathion 5EC	44.8	2	175	104.85	793.79

**Table 3:** The amount of each insecticide (mixed with Nu-Lure Insect Bait) applied to roosting plants of *Zeugodacus cucurbitae* in the perimeter of cucurbit crop fields per application during field trials that tested each insecticide separately. The approximate area covered by the treated spots is the area of cucurbit crops inside the surrounding treated roosting plants.

Insecticide Product	Trial Location (Year)	Total Volume of Spray	Water	Nu-Lure	Amount of Insecticide Product	Ammonium Acetate (% of Final Mixture)	Vol. per Spot Spray	No. of Spot Sprays	Approx. Area Covered by Treated Spots	g of Active Ingredient/Acre
Agri-Mek SC	Maui commercial farm (2020)	0.95 gal (3.6 L)	0.75 gal (2.83 L)	24 fl oz (0.71 L)	1.758 fl oz (52 mL)	36 g (1%)	3 fl oz (90 mL)	40	5 acres (2 hectares)	0.872
	Oahu commercial farm (2020)	8 gal (30.28 L)	6.31 gal (23.88 L)	201.7 fl oz (5.96 L)	14.77 fl oz (437 mL)	151.4 g (0.5%)	3 fl oz (90 mL)	336	96 acres (39 hectares)	0.382
	Maui research station (2021)	1.43 gal (5.4 L)	1.32 gal (4.98 L)	14.2 fl oz (0.415 L)	0.25 fl oz (7.4 mL)	54 g (1%)	3 fl oz (90 mL)	60	6.3 acres (2.5 hectares)	0.099
Mustang Maxx	Maui commercial farm (2020)	1.43 gal (5.4 L)	1.12 gal (4.25 L)	36 fl oz (1.065 L)	3 fl oz (89 mL)	54 g (1%)	3 fl oz (90 mL)	60	5 acres (2 hectares)	1.706
	Maui research station (2021)	1.43 gal (5.4 L)	1.31 gal (4.97 L)	13.8 fl oz (0.408 L)	0.52 fl oz (15.5 mL)	54 g (1%)	3 fl oz (90 mL)	60	6.3 acres (2.5 hectares)	0.236
Malathion 5EC	Maui commercial farm (2020)	1.43 gal (5.4 L)	1.00 gal (3.77 L)	36 fl oz (1.065 L)	19.2 fl oz (568 mL)	54 g (1%)	3 fl oz (90 mL)	60	5 acres (2 hectares)	68.061
	Maui research station (2021)	1.43 gal (5.4 L)	1.29 gal (4.90 L)	14.0 fl oz (0.414 L)	3.11 fl oz (92 mL)	54 g (1%)	3 fl oz (90 mL)	60	6.3 acres (2.5 hectares)	8.749

**Table 4:** The amount of each insecticide (mixed with Nu-Lure Insect Bait) applied to roosting plants of *Zeugodacus cucurbitae* in the perimeter of cucurbit crop fields per application during the insecticide rotation trial on the Maui commercial farm. The approximate area covered by the treated spots is the area of cucurbit crops inside the surrounding treated roosting plants.

Insecticide Product	Application Dates	Total Volume of Spray	Water	Nu-Lure	Amount of Insecticide Product	Ammonium Acetate (% of Final Mixture)	Vol. Per Spot Spray	No. of Spot Sprays	Approx. Area Covered by Treated Spots	g of Active Ingredient/Acre
GF-120	29 April 21, 6 May 21, 1 July 21, 8 July 21	1.43 gal (5.4 L)	0.86 gal (3.24 L)	-	0.57 gal (2.16 L)	-	3 fl oz (90 mL)	40	5 acres (2 hectares)	0.104
Agri-Mek SC	13 May 21, 20 May 21, 15 July 21, 22 July 21	1.43 gal (5.4 L)	1.32 gal (4.98 L)	14.2 fl oz (0.415 L)	0.25 fl oz (7.4 mL)	54 g (1%)	3 fl oz (90 mL)	60	5 acres (2 hectares)	0.124
Mustang Maxx	3 June 21, 10 June 21	1.43 gal (5.4 L)	1.31 gal (4.97 L)	13.8 fl oz (0.408 L)	0.52 fl oz (15.5 mL)	54 g (1%)	3 fl oz (90 mL)	60	5 acres (2 hectares)	0.297
Malathion 5EC	17 June 21, 24 June 21	1.43 gal (5.4 L)	1.29 gal (4.90 L)	14.0 fl oz (0.414 L)	3.11 fl oz (92 mL)	54 g (1%)	3 fl oz (90 mL)	60	5 acres (2 hectares)	11.024

**Table 5:** Yield of marketable zucchini fruits (numbers of 4-gallon buckets filled) and *Zeugodacus cucurbitae*-infested fruits during the insecticide rotation trial at the Maui commercial farm. The data was collected by the farm manager.

		Treated Area			Control Area		
Zucchini Harvest Date	Insecticide Treatment the Week Prior to Harvest	No. Buckets of Marketable Fruits	No. Buckets of Infested Fruits	Marketable Harvest (%)	No. Buckets of Marketable Fruits	No. Buckets of Infested Fruits	Marketable Harvest (%)
29 April 2021	No treatment	-	-	-	-	-	-
6 May 2021	GF-120	24	23	51%	28	25	53%
13 May 2021	GF-120	35	28	55%	26	21	55%
20 May 2021	Agri-Mek SC	48	20	71%	32	24	57%
27 May 2021	Agri-Mek SC	47	8	85%	27	21	56%
3 June 2021	Mustang Maxx	45	3	94%	17	12	59%
10 June 2021	Mustang Maxx	46	3	94%	No harvest	No harvest	-
17 June 2021	Malathion 5EC	42	1	98%	23	14	62%
24 June 2021	Malathion 5EC	27	0.5	98%	24	8	75%
1 July 2021	GF-120	23	1	96%	16	4	80%

## Data Availability

The data presented in this study are available on request from the corresponding author.

## Supplementary Materials

The following supporting information can be downloaded at: https://www.mdpi.com/article/10.3390/insects13070629/s1. Table S1: Total number of *Zeugodacus cucurbitae*, including females (F) and males (M), caught weekly in each trap during the insecticide rotation trial on the Maui commercial farm. The dates refer to the date that traps were collected and counted; the insecticide was applied one week prior to the trap count date.
