# Supplementary material for: Field Tests of Three Alternative Insecticides with Protein Bait for the Development of an Insecticide Rotation Program to Control Melon Flies, Zeugodacus cucurbitae (Coquillett) (Diptera: Tephritidae)
Source: Insects. 2022 Jul 14;13(7):629. doi: 10.3390/insects13070629 (PMC9323022; doi:10.3390/insects13070629)
Supplement: Supplementary file 1 [file insects-13-00629-s001.zip › insects-1796662-supplementary.pdf]

Table S1. Total number of *Zeugodacus cucurbitae*, including females (F) and males (M), caught weekly in each trap during the insecticide rotation trial on the Maui commercial farm. The dates refer to the date that traps were collected and counted; the insecticide was applied one week prior to the trap count date.

Trap count date	Insecticide treatment the week prior to trap count	Control Area						Treated Area								
		Trap 1			Trap 2			Trap 3			Trap 4			Trap 5		
		Total	F	M	Total	F	M	Total	F	M	Total	F	M	Total	F	M
04/29/2021	Pre-treatment	409	225	184	-	-	-	2388	1114	1274	5060	2760	2300	1215	660	555
05/06/2021	GF-120	713	399	314	274	164	110	199	82	117	879	404	475	771	293	478
05/13/2021	GF-120	714	357	357	889	587	302	57	25	32	1532	552	980	172	69	103
05/20/2021	Agri-Mek SC	644	303	341	1455	757	698	31	13	18	1249	487	762	105	38	67
05/27/2021	Agri-Mek SC	298	182	116	637	420	217	26	16	10	1389	722	667	140	66	74
06/03/2021	Mustang Maxx	243	139	104	345	252	93	128	75	53	1179	733	446	218	103	115
06/10/2021	Mustang Maxx	230	159	71	132	98	34	21	2	19	1124	495	629	408	126	282
06/17/2021	Malathion 5EC	255	138	117	37	33	4	28	16	12	944	453	491	529	100	429
06/24/2021	Malathion 5EC	352	253	99	25	18	7	61	37	24	1237	730	507	402	117	285
07/01/2021	GF-120	330	132	198	148	76	72	187	99	88	1206	579	627	333	77	256
07/08/2021	GF-120	928	418	510	233	163	70	448	139	309	714	236	478	540	232	308
07/15/2021	Agri-Mek SC	412	239	173	97	47	50	315	145	170	872	305	567	1201	192	1009
07/22/2021	Agri-Mek SC	314	210	104	90	65	25	266	146	120	555	322	233	2029	467	1562
07/29/2021	No treatment	880	607	273	187	133	54	479	280	199	380	232	148	1293	272	1021
08/05/2021	No treatment	645	413	232	181	145	36	278	164	114	250	158	92	779	280	499
